# Supplementary material for: G protein-specific mechanisms in the serotonin 5-HT2A receptor regulate psychosis-related effects and memory deficits
Source: Nat Commun. 2024 May 29;15:4307. doi: 10.1038/s41467-024-48196-2 (PMC11137019; doi:10.1038/s41467-024-48196-2)
Supplement: Supplementary file 1 — Supplementary Information [file 41467_2024_48196_MOESM1_ESM.pdf]

Supplementary Information

G protein-specific mechanisms in the serotonin 5-HT2A receptor regulate psychosis-related effects and memory deficits

Elk Kossatz[1#], Rebeca Diez-Alarcia[2,3,4#], Supriya A. Gaitonde[5], Carla Ramon-Duaso[6], Tomasz Maciej Stepniewski[7,8], David Aranda-Garcia[7,13], Itziar Muneta-Arrate[2,3], Elodie Tepaz[5], Suwipa Saen-Oon[9], Robert Soliva[9], Aida Shahraki[10], David Moreira[11,12], Jose Brea[11,12], Maria Isabel Loza[11,12], Rafael de la Torre[1], Peter Kolb[10], Michel Bouvier[5], J. Javier Meana[2,3,4], Patricia Robledo[1]* and Jana Selent[7,13]*

[1]*Integrative Pharmacology and Systems Neuroscience Research Group, Hospital del Mar Research Institute, Barcelona, Spain*
[2]*Department of Pharmacology, University of the Basque Country/Euskal Herriko Unibertsitatea, Leioa, Bizkaia, Spain*
[3]*Centro de Investigación Biomédica en Red de Salud Mental CIBERSAM, Spain*
[4]*Biocruces Bizkaia Health Research Institute, Barakaldo, Bizkaia, Spain*
[5]*Department of Biochemistry and Molecular Medicine, Institute for Research in Immunology and Cancer (IRIC), Université de Montréal, Montréal, Québec H3T 1J4, Canada*
[6]*Cell-type mechanisms in normal and pathological behaviour Research Group, IMIM-Hospital del Mar Medical Research Institute, Barcelona, Spain*
[7]*Research Programme on Biomedical Informatics (GRIB), Hospital del Mar Research Institute, Barcelona, Spain*
[8]*InterAx Biotech AG, PARK InnovAARE 5234 Villigen Switzerland*
[9]*NBD NOSTRUM BIODISCOVERY, Av. de Josep Tarradellas, 8-10, 3-2, 08029, Barcelona, Spain*
[10]*Pharmaceutical Chemistry, University of Marburg, Marbacher Weg 8, Marburg 35037, Germany*
[11]*Innopharma Drug Screening and Pharmacogenomics Platform. BioFarma research group. Center for Research in Molecular Medicine and Chronic Diseases (CiMUS). Department of Pharmacology, Pharmacy and Pharmaceutical Technology. University of Santiago de Compostela, Santiago de Compostela, Spain*
[12]*Health Research Institute of Santiago de Compostela (IDIS), University Hospital of Santiago de Compostela (SERGAS), Trav. Choupana s/n, 15706 Santiago de Compostela, Spain*
[13]*Department of Medicine and Life Sciences, Pompeu Fabra University, Barcelona, Spain*

[#]Authors contributed equally
*Correspondence to: Jana Selent and Patricia Robledo

PRBB
Calle Dr. Aiguader 88
Barcelona 08003, SPAIN
probledo@imim.es; jana.selent@upf.edu
Tel: +34 93 316 0455

Table of Content

1. G protein coupling profile in WT and 5-HT_{2A}R KO mice brain tissue
2. β-Arrestin implication
3. Previous findings on cognitive skills via the 5-HT_{2A}R
4. Methodology
5. Supplemental Figures
6. Supplemental Tables
7. References

1. G protein coupling profile in WT and 5-HT_{2A}R KO mice brain tissue

To further demonstrate the role of 5-HT_{2A}R in the observed effects, a single submaximal concentration of the drugs (10 µM) was used for [^{35}S]GTPγS binding experiments coupled to immunoprecipitation of $G_{\alpha i1}$, $G_{\alpha i2}$, $G_{\alpha i3}$ and $G_{\alpha q/11}$ of G_{α} subunit subtypes of heterotrimeric G proteins in membrane homogenates from the brain cortex of WT and 5-HT_{2A}R KO mice (Figure 2, Figure S2, Table S1). These experiments confirmed the signaling profile described for the four compounds in postmortem human PFC, with small differences. In WT mice, Met-I modulated the activity of all the studied G_{α} subunit subtypes, with the exception of $G_{\alpha i2}$. This drug increased the basal activity of $G_{\alpha i3}$, and $G_{\alpha q/11}$, while it reduced the basal activity of $G_{\alpha i1}$. All of these effects were absent in 5-HT_{2A}R KO mice (Figure S2, Table S1). In WT mice, Nitro-I showed statistically significant activation of $G_{\alpha i1}$ and $G_{\alpha q/11}$. While stimulation of $G_{\alpha i3}$ was still significant in KO brain tissue, there was no stimulation of $G_{\alpha i1}$ nor $G_{\alpha q/11}$ by Nitro-I (Figure S2, Table S1). OTV1 reduced the basal activity of $G_{\alpha i1}$ in WT mice, and this inverse agonism was absent in 5-HT_{2A}R KO samples. On the other hand, this drug increased the [^{35}S]GTPγS binding to $G_{\alpha i2}$, $G_{\alpha i3}$ and $G_{q/11}$ in WT mice, while a significant stimulation of $G_{\alpha i2}$ was observed only in KO mice (Figure S2, Table S1). OTV2 increased the activation of all the tested G subunit subtypes in WT mice. When the same experiments were carried out with membranes from 5-HT_{2A}R KO mice, no stimulation of $G_{\alpha i1}$, $G_{\alpha i3}$ nor $G_{\alpha q/11}$ was observed, while a statistically significant increase of [^{35}S]GTPγS binding to $G_{\alpha i2}$ was detected (Figure S2, Table S1).

2. β-Arrestin implication in pro-psychotic behavior

Past *in vitro* and *in vivo* studies have shown contradictory results on the role of β-arrestins in HTR. For example, experiments carried out with β-arrestin 2 KO mice found that while the non-hallucinogenic 5-HT and its metabolic precursor 5-HTP require β-arrestin 2 for the expression of HTR, hallucinogens like DOI and N-methyltryptamines do not (Schmid et al., 2008; Schmid and Bohn, 2010). Furthermore, HTR elicited by N-methyltryptamines are enhanced in β-arrestin 2 KO mice (Schmid and Bohn, 2010), and the HTR induced by DOI is abrogated in PSD95-null mice (Abbas et al., 2009). However, recent works suggest that LSD's psychedelic drug-like actions appear to require β-arrestin 2 (Rodriguiz et al., 2021), and the role of an interaction of $G_{i/o}$ subunits with β-arrestins cannot be discarded (Smith et al., 2021). In our present work, we found that OTV1 showed almost no efficacy/potency for β-arrestins 1 and 2 recruitment in cells, and did not elicit HTR. Our data show that Met-I, Nitro-I and OTV2 exhibited similar efficacy/potency for recruiting β-arrestins 1/2 in BRET assays, but only Met-I and OTV2 elicited HTR in mice, suggesting that activation of β-arrestins may be necessary but not sufficient for the appearance of HTR.

3. Previous findings on cognitive skills via the 5-HT$_{2A}$R

Previous studies have shown that the effects of 5-HT$_{2A}$R ligands on cognitive functioning vary depending on the different tasks used. For example, the phenethylamine-derived hallucinogen TCB-2, a potent, high-affinity 5-HT$_{2A}$R ligand was shown to enhance long-term novel object recognition memory in mice (Zhang et al., 2013), but it significantly impaired hippocampal spatial memory in the Morris water maze task (Zhang and Stackman, 2015), as did the psychedelic tryptamine psilocin (Rambousek et al., 2014). On the other hand, the psilocin precursor, psilocybin, increased the extinction of fear memories (Catlow et al., 2013), and the hallucinogen DOI was found to reduce memory retrieval in a non-operant response in rats (Meneses, 2007). These contrasting results could also be due to differences in the functional selectivity of the compounds studied, but surprisingly, no studies have been published looking at the involvement of different G protein pathways in these effects.

4. Methodology

Ligand binding studies

5-HT$_{2A}$R competition binding experiments were carried out in a polypropylene 96-well plate. In each well was incubated 70 µg of membranes from CHO-5-HT 2A cell line prepared in our laboratory (Lot: A006/10-03-2020, protein concentration=5134 µg/ml), 1 nM [^{3}H]Ketanserin (47.3 Ci/mmol, 1 mCi/ml, Perkin Elmer NET791250UC) and compounds studied and standard. Non-specific binding was determined in the presence of Methysergide 1 µM (Sigma M137). The reaction mixture (Vt : 250 µl/well) was incubated at 37°C for 30 min, 200 µl was transferred to GF/B 96-well plate (Millipore, Madrid, Spain) pretreated with 0.5% of PEI and treated with binding buffer (Tris-HCl 50 mM, pH=7.4), after was filtered and washed six times with 250 µl wash buffer (Tris-HCl 50 mM, pH=6.6), before measuring in a microplate beta scintillation counter (Microbeta Trilux, PerkinElmer, Madrid, Spain).

Bioluminescence resonance energy transfer assay

HEK-293 cells were transfected with plasmids encoding for the 5-HT$_{2A}$R receptor, the respective G protein subunits and the ebBRET biosensor pair using linear polyethylenimine (3:1 PEI:DNA), distributed (3.5 x 10^4 cells/well) in 96-well white microplates (Greiner Bio-One) and maintained at 37 °C with 5% CO_2 for 48 h before conducting BRET experiments. On the day of the BRET experiment, cells were washed with phosphate-buffered saline (PBS), treated

with Tyrode's buffer (140 mM NaCl, 2.7 mM KCl, 1 mM $CaCl_2$, 12 mM $NaHCO_3$, 5.6 mM D-glucose, 0.5 mM $MgCl_2$, 0.37 mM NaH_2PO_4, 25 mM HEPES [pH 7.4]), and allowed to equilibrate at 37 °C for at least 15 min. Cells were then treated with an increasing concentration of the test compounds, incubated for 5 min after which the BRET substrate Prolume Purple [methoxy e-Coelenterazine (Me-O-e-CTZ)] (Nanolight) was added. After an additional incubation of 6 min, the BRET signal (agonist mode) was measured using the Spark® multimode microplate reader (Tecan) equipped with a dedicated PMT - single photon counting Multi-color (acceptor, 515 ± 20 nm and donor, 400 ± 70 nm filters).

Raw BRET data is the ratio of the light intensity emitted by the acceptor over the light intensity emitted by the donor (rGFP/RlucII). Ligand-promoted BRET (ΔBRET was calculated by subtracting the BRET ratio of the compounds from that of the vehicle condition. The data were then normalized with respect to the maximal response of 5-HT. The transduction coefficients ($\log(\tau/K_A)$) of the compounds at each pathway were extrapolated from data analysis in PRISM 9.2.0.

Calculation of the bias factor

The bias of the compounds towards the different signaling pathways downstream of the 5-$HT_{2A}R$ receptor was calculated by the method by Kenakin and Christopoulos (Kenakin and Christopoulos, 2013; Nagi and Pineyro, 2016) from the transduction coefficient ($\log \tau/K_A$) or (Log R) generated from the concentration-response curves on GraphPad Prism using the Nonlinear Regression parameter "New Operational Model with TauKa Ratios". The ($\log \tau/K_A$) value for the compounds for a given pathway was first subtracted from that of 5-HT to give the $\Delta(\log \tau/K_A)$, which was then subtracted from the $\Delta(\log \tau/K_A)$ obtained for a second signaling pathway to give the $\Delta\Delta(\log \tau/K_A)$ such that it is a positive value. The antilog of $\Delta\Delta(\log \tau/K_A)$ gives the bias factor.

Prefrontal cortex membranes preparation

Membrane-enriched fractions (P2) from human and mouse samples were prepared as previously described (Diez-Alarcia et al., 2016). For postmortem human brain studies, samples from six different subjects were processed each time, and two different pools of samples were used for the whole study. Thus, samples from 12 different subjects (10 males and 2 females) with ages between 29–90 years were included. The postmortem delay between death and storage of the samples ranged from 4 to 12 h, and the storage time between sampling and experiments ranged from 48 to 10 months. In the case of studies with

mouse prefrontal cortex samples from five mice were simultaneously processed each time, and again two different pools of samples were used.

Antibody-capture [^{35}S]GTPγS binding scintillation proximity assay (SPA)

[^{35}S]GTPγS binding was performed in a buffer containing 0.4 nM [^{35}S]GTPγS, 11 μg of protein/well and 50/100 μM GDP depending on the studied subunit (Table S2). After [^{35}S]GTPγS binding, immunoprecipitation with specific antibodies for each G-protein subunit ($G_{\alpha i1}$, $G_{\alpha i2}$, $G_{\alpha i3}$ and $G_{\alpha q/11}$) and protein A polyvinyltoluene SPA beads was carried out. Mouse monoclonal antibodies against $G_{\alpha i1}$ (sc-56536), $G_{\alpha i2}$ (sc-13534), and $G_{\alpha q/11}$ (sc-515689) proteins were obtained from Santa Cruz Biotechnologies (USA). Rabbit polyclonal anti-$G_{\alpha i3}$-protein (ABIN6258933) was obtained from Antibodies online. See Table S2 for further details. The specificity of the antibodies *vs* the different recombinant G proteins and brain tissue has been previously demonstrated (Diez-Alarcia et al., 2021).

Plates were then centrifuged and bound radioactivity was detected on a MicroBeta TriLux scintillation counter (Perkin Elmer, Madrid, Spain). Non-specific binding was defined as the remaining [^{35}S]GTPγS binding in the presence of 100 μM unlabeled GTPγS. In order to confirm the 5-HT_{2A}R involvement in the observed effects, one-point concentration experiments (10 μM) in the absence and presence of the antagonist MDL-11,939 (1 μM) were carried out with postmortem human brain samples, and also in 5-HT_{2A}R KO and WT mice brain samples.

Western Blots

Mice were sacrificed 24 h after the ICV infusions and the cortex was dissected and stored at -80ºC until processing. Brain homogenates were processed as previously described (Urigüen et al., 2009) with minor modifications. Briefly, brain samples (~10 mg) were thawed at 4°C and homogenized in 30 v/w of cold homogenization buffer (150 nM NaCl, 1% TX-100, 10% glycerol, 1 mM EDTA, and 50 mM Tris–HCl buffer, pH 7.5, containing protease and phosphatase inhibitors) using a Potter (2 x 20 pulses). Samples were incubated in ice for 30 min, centrifuged for 10 min at 20,000xg (4°C), and supernatants kept. Protein content was determined using a Bio-Rad DC Protein Assay Kit with BSA as standard. Samples were then diluted in a homogenization buffer until reaching a concentration of 2 mg protein/ml, aliquoted, and kept at −70°C. Western blot was performed as previously described (Ibarra-Lecue et al., 2020) with minor modifications. Commercial Laemmli buffer (95% v/v) and β-mercaptoethanol (5% v/v) were added to each sample. Samples were denatured (95°C for 5 min), loaded (12 μg), and resolved to SDS-PAGE onto polyacrylamide gel (12 %). Samples were then

transferred to nitrocellulose membranes (Amersham TM Protran TM 0.45 μm NC). Nitrocellulose membranes were blocked (5% nonfat dry-milk) in phosphate-buffered saline (PBS) followed by overnight incubation with primary antibodies (4°C). Specific antibodies against each G protein subunit ($G_{\alpha i1}$, $G_{\alpha i3}$ and $G_{\alpha q/11}$) and β-actin (polyclonal rabbit anti-β-actin, ab8227, AbCam) were used. Antibodies against G_{α}-protein subunit were the same used for Antibody-capture [^{35}S]GTPγS binding scintillation proximity assay (SPA) (Table S2) although used at different dilutions ($G_{\alpha i1}$ 1:200, $G_{\alpha i3}$ 1:300 and $G_{\alpha q/11}$ 1:250). Incubation with fluorescent anti-IgG secondary antibodies (Alexa 680 Goat anti-mouse, Molecular Probes; DyeLight800 611.732.127, BiomolGmbH) was performed at room temperature (1 h). Immunoreactivity was detected using an Odyssey Infrared Imaging System (LI-COR Biosciences, Lincoln, NE, USA) and quantified (as integrated intensity values) using ImageJ. The immunoreactivity value of the target proteins was corrected by the corresponding value of β-actin; and then, calculated as a percentage of a standard sample loaded in every single gel to control for interassay variability. Representative images of immunoblots can be found as insets in Figure S4.

Mice genotyping

Homozygous 5-HT_{2A}R knockout (KO) mice and wild-type (WT) littermates were used, and to confirm the genotype, we performed PCR analysis from the ear DNA using the following primers: 5'-TGGGCGAGTTTACGGGTTGTTA-3' (5-HT_{2A}R KO forward); 5'-GTGGCTCCTCTGGCTGCTTTCT-3' (WT forward); 5'-CTGTGGGATTTTCTTTCTGCTT-3' (5-HT_{2A}R KO and WT reverse).

Intracerebroventricular Surgery and Infusion

Mice were anesthetized with a mixture of ketamine (75 mg/kg, i.p.) and medetomidine (1 mg/kg, i.p.) at a volume of 0.1 ml/10 g of body weight and placed in a stereotaxic apparatus (RWD Life Science Co.). Unilateral cannulae (26 G; 9 mm length) were implanted in the left lateral ventricle (AP -0.2 mm; ML +1.0 mm; DV -2.5 mm from bregma), and fixed to the skull with dental acrylic. After surgery, mice were individually housed. One week after surgery, ICV infusions were performed using a 10 µl Hamilton® microsyringe attached to a micro infusion pump (Cole-Parmer Instrument Co.). The infusion was conducted at a constant rate of 1 µl/min, connecting the micro syringe to the ICV cannula through a polyethylene tube (PE-20, Plastics One). To prevent drug reflux, the tube was removed from the cannula 1 min after the infusion. At the end of the experiments, animals received an ICV infusion of 1% methylene blue solution to confirm the correct cannula placement.

The novel object recognition (NOR) test.

This test is performed in 3 days. On day 1, 24 h before ICV infusions, mice were habituated to the empty maze for 9 min. On day 2 (15 min before ICV infusion), mice were trained in the maze for 9 min with two identical objects placed on each arm of the maze. On day 3 (24 h after ICV infusion), mice were tested in the maze for 9 min, where one of the familiar objects was replaced by a novel object. The total time spent exploring the novel and the familiar objects was recorded. A discrimination index (DI) was calculated as the difference between the time spent exploring either the novel (Tn) or familiar (Tf) object divided by the total time exploring both objects (DI = (Tn − Tf)/(Tn + Tf)). In this test, a higher discrimination index indicates better memory retention (Da Cruz et al., 2020; Viñals et al., 2015). DI values above 0.3 were considered to reflect memory retention for the familiar object.

Sucrose Preference test

Prior to beginning the test, mice were habituated to the presence of two volumetric pipettes (instead of bottles) of drinking water in their home cage for 3 days. On the fourth day, mice received ICV infusions and had access to two volumetric pipettes, one containing drinking water and the other containing 1% sucrose for 3 days, and the volume of solution intake was measured daily. The position of the pipettes was alternated daily to avoid place preference bias. Sucrose preference (SP) was calculated as a percentage of the volume of sucrose intake over the total volume of solution intake and averaged over the 3 days of testing (SP = V (sucrose solution)/ [V (sucrose solution) + v (water)] x 100%. In this test, a reduced preference for sweet solution represents anhedonia.

5. Supplemental Figures

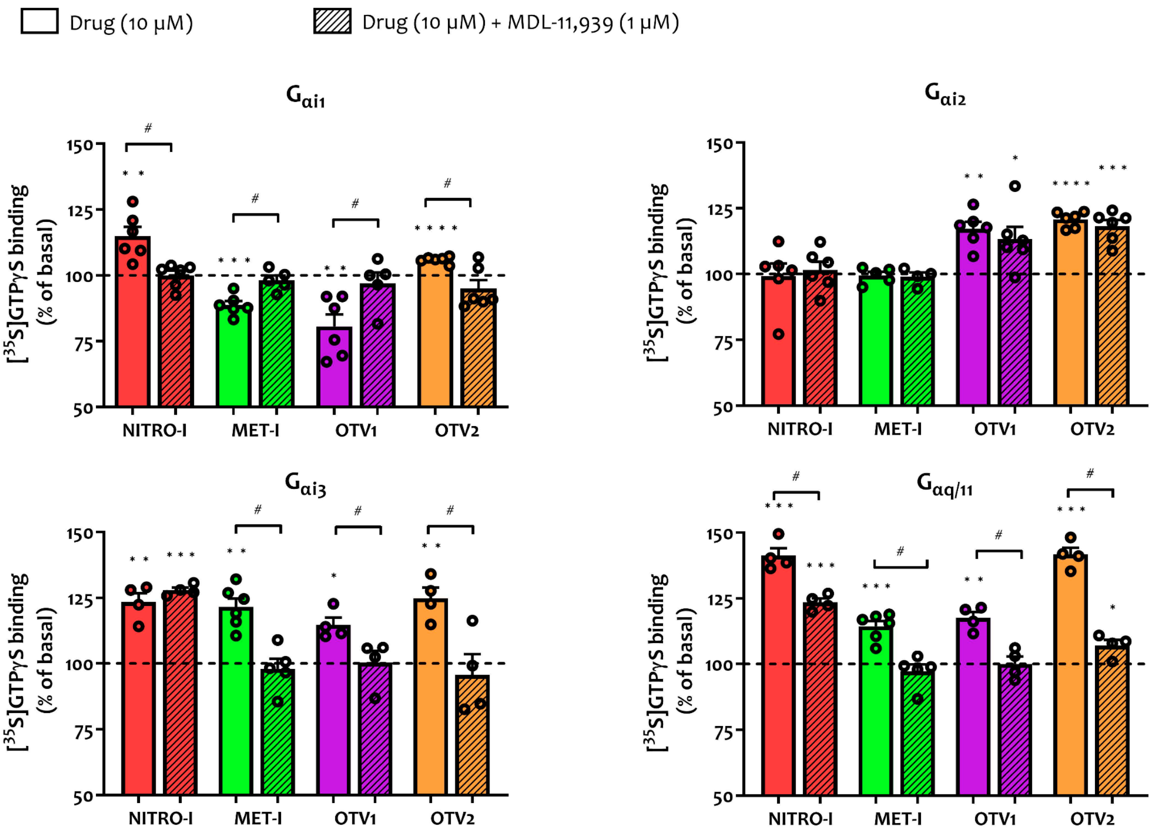

Figure S1. G protein activity in postmortem human brain tissue. Modulation of specific [^{35}S]GTPγS binding to $G_{\alpha i1}$, $G_{\alpha i2}$, ${}_{\alpha}G_{i3}$, and $G_{\alpha q/11}$ proteins by 10 μM Nitro-I, Met-I, OTV1 and OTV2 in the human prefrontal cortex. Basal values of specific [^{35}S]GTPγS binding to the different G_{α} proteins are expressed as 100% and stimulatory/inhibitory effects on the respective basal are shown. Individual dots represent different assays (4-6) carried out for each subunit/condition performed in duplicate or triplicate. *$p<0.05$, **$p<0.01$, ***$p < 0.001$ *vs* 100%; $^{\$}p<0.05$ *vs* incubation in the presence of 1 μM MDL-11,939 (striped bars) (*t*-test).

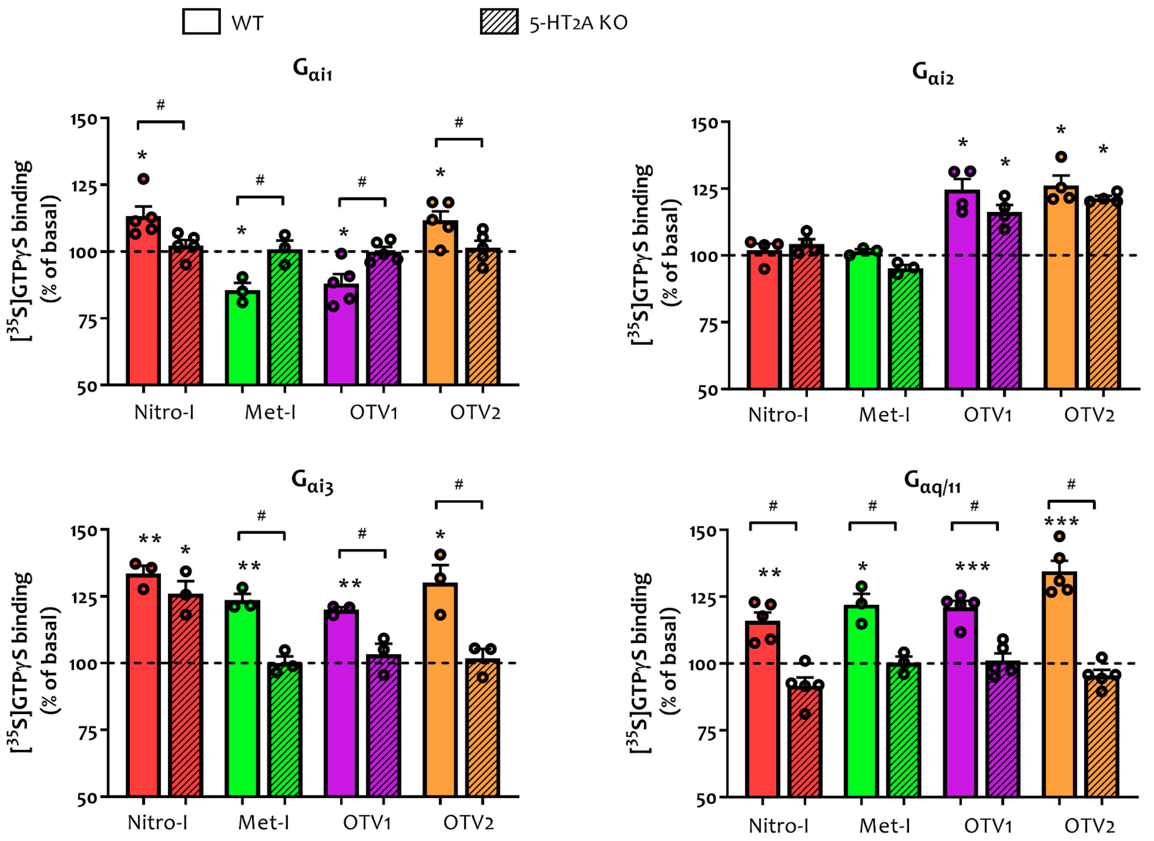

Figure S2. G protein activity in 5-HT$_{2A}$R KO and WT mice brain tissue. Modulation of specific [^{35}S]GTPγS binding to G$_{\alpha i1}$, G$_{\alpha i2}$, $_{\alpha}$G$_{i3}$, and G$_{\alpha q/11}$ proteins by 10 μM Nitro-I, Met-I, OTV1 and OTV2 in brain cortex membranes from 5-HT$_{2A}$R KO and WT mice. Basal values of specific [^{35}S]GTPγS binding to the different G$_{\alpha}$ proteins are expressed as 100% and stimulatory/inhibitory effects on the respective basal are shown. Individual dots represent the different assays (3-5) for each subunit performed in duplicate or triplicate. *p<0.05, **p<0.01, ***p< 0.001 *vs* 100%; $^{\$}$p<0.05 KO *vs* WT (striped bars) (*t*-test).

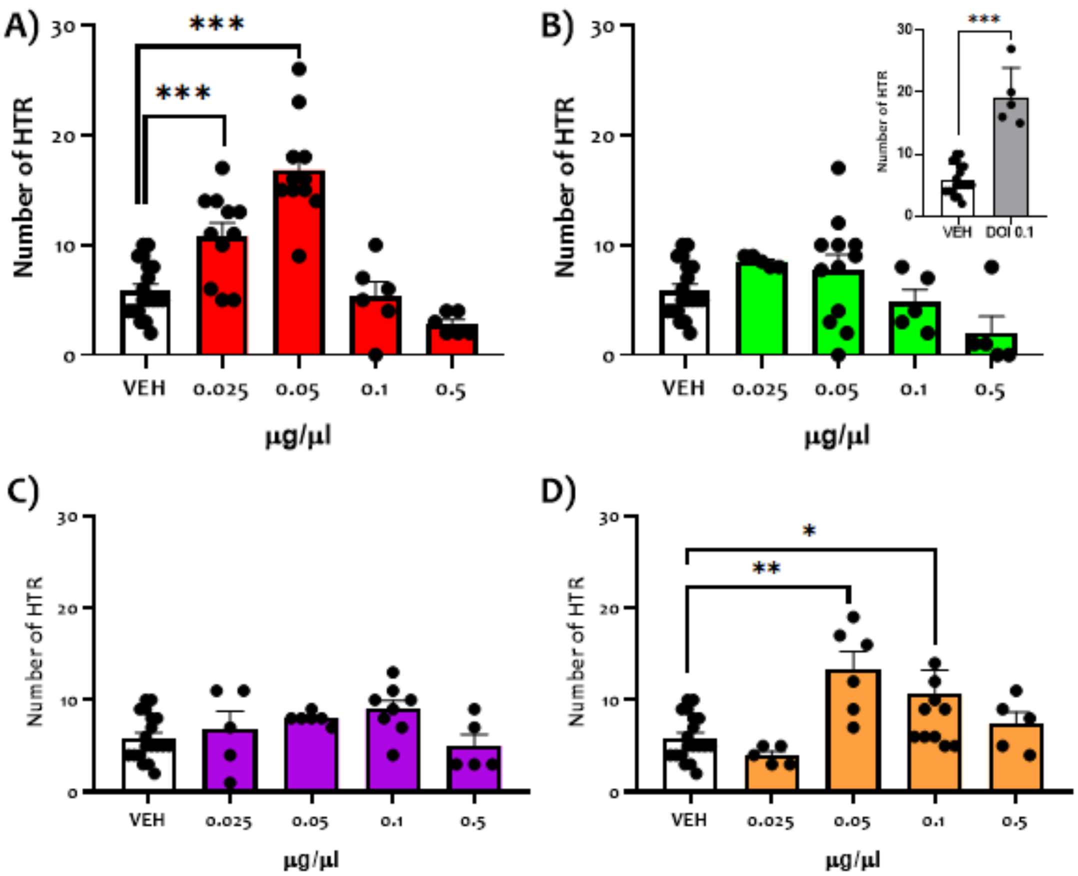

Figure S3. Head twitch response (HTR) following ICV administration of different doses of (A) Nitro-I, (B) Met-I, (C) OTV1, and (D) OTV2 or vehicle (VEH), and DOI as a control (inset). Nitro-I significantly increased HTR with respect to VEH at the doses of 0.025 and 0.05 µg/µl, while OTV2 significantly increased HTR at the doses of 0.05 and 0.1 µg/µl. Met-I and OTV1 did not modulate HTR at any dose tested. DOI significantly increased HTR with respect to VEH (inset). The data represent mean ± SEM. The number of independent samples (n) corresponds to the individual points in the graph for each dose. Effect of DOI vs. VEH (two-tailed unpaired t-test). Dose-response (one-way ANOVAs followed by Fisher's post-hoc test). *p<0.05, **p<0.01, ***p<0.001 *vs* VEH.

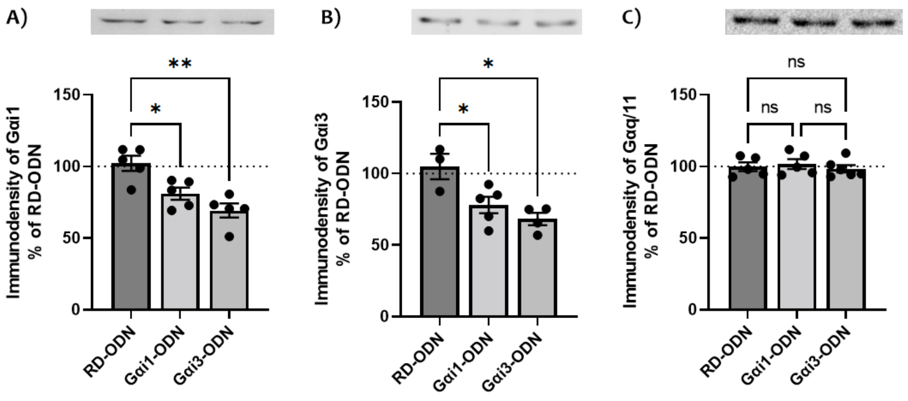

Figure S4. Immunodensity of $G_{\alpha i1}$, $G_{\alpha i3}$ and $G_{\alpha q/11}$ after ODN treatment. Relative expression levels of (A) $G_{\alpha i1}$, (B) $G_{\alpha i3}$ and (C) $G_{\alpha q/11}$ proteins in total homogenates of prefrontal cortex samples from mice treated with OTV2 and with antisense oligonucleotides inhibiting $G_{\alpha i1}$ ($G_{\alpha i1}$-ODN), $G_{\alpha i3}$ ($G_{\alpha i3}$-ODN) or a control random sequence (RD), and representative western blot images (inset). In mice chronically treated with $G_{\alpha i1}$- or $G_{\alpha i3}$-ODNs the immunodensitometric signal of both $G_{\alpha i1}$ ($F(2, 12)=12.04$, $p<0.001$) and $G_{\alpha i3}$ ($F(2, 9)=7.92$, $p<0.01$) appeared clearly reduced compared to that observed in animals treated with RD-ODN, while no change was observed for the expression levels of $G_{\alpha q/11}$ ($F(2, 13)=0.323$, $p=0.730$). The data represent mean ± SEM. The number of independent samples (n) corresponds to the individual points in the graph for each condition. The data were analyzed by one-way ANOVA followed by *post hoc* Bonferroni for multiple comparisons. *p< 0.05, **p < 0.01 *vs* RD-ODN. ns: non-significant.

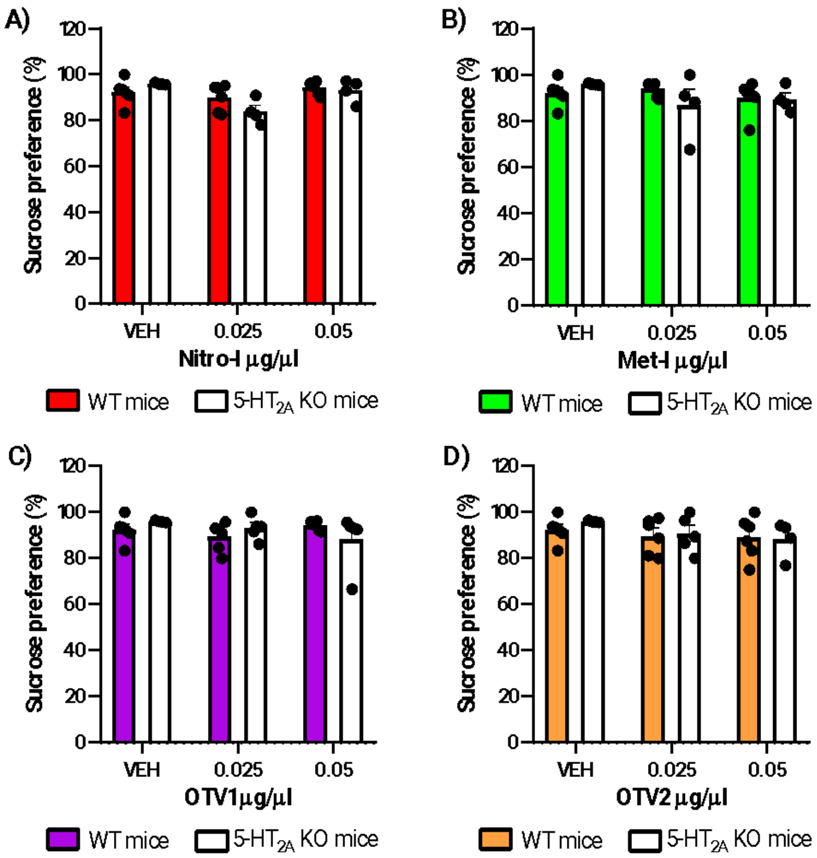

Figure S5. Sucrose preference test in wild-type (WT) and 5-HT_{2A}R knockout (KO) mice following ICV administration of (A) Nitro-I, (B) Met-I, (C) OTV1, and (D) OTV2 or vehicle (VEH). None of the 4 compounds elicited depression-like behavior at 0.025 or 0.05 µg/µl doses in either genotype. The data represent mean ± SEM.The number of independent samples (n) corresponds to the individual points in the graph for each dose.

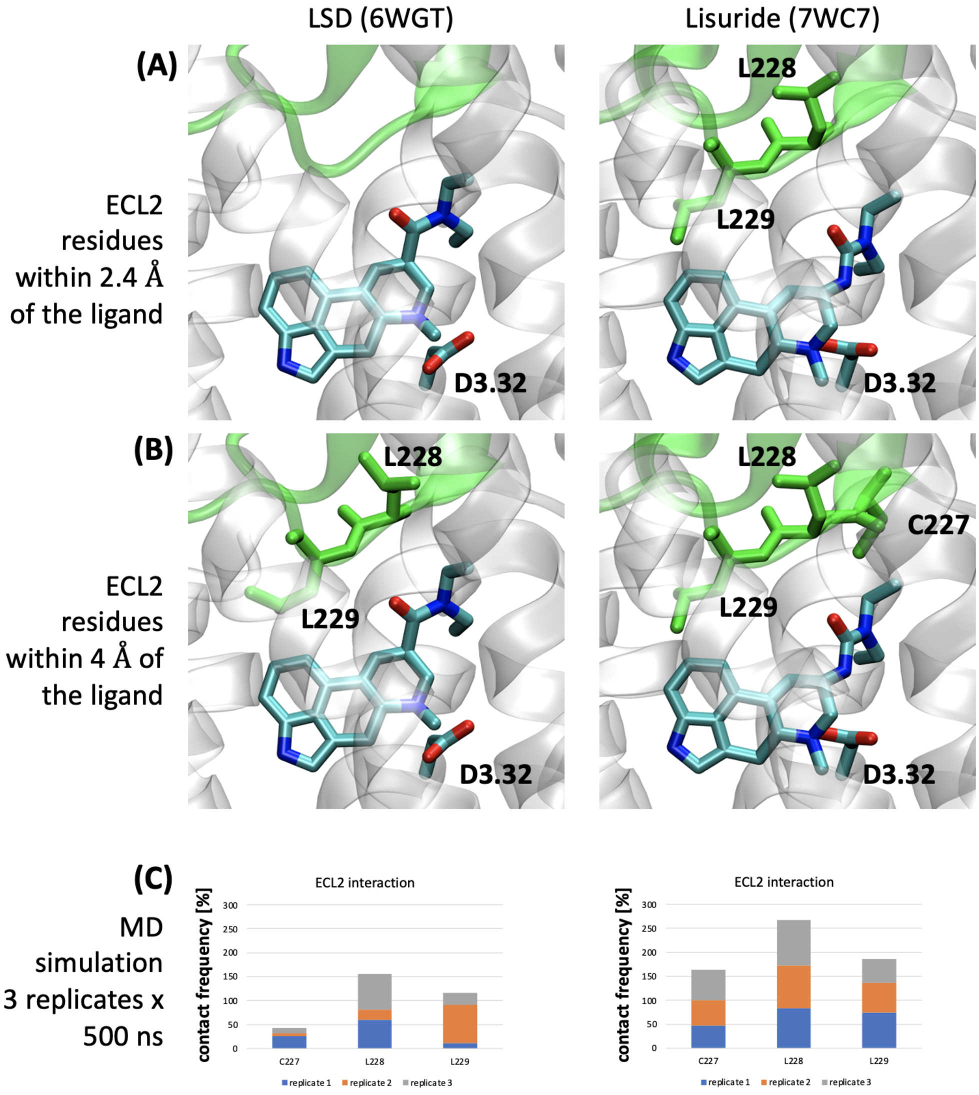

Figure S6. Differential ECL2 binding properties for the experimentally solved complexes of LSD and lisuride bound to the 5-HT_{2A}R. (A) The non-hallucinogenic lisuride (PDB 7WC7) seems to be closely packed against the ECL2 as two residues in the ECL (L228, L229) are found within 2.4 Å distance from the ligand. In contrast, no ELC2 residues are found for the hallucinogenic LSD (PDB 6WGT) within this distance. (B) Increasing the distance to 4 Å, three ECL2 residues (L228, L229, C230) are detected for lisuride whereas only two for LSD (L228, L229). (C) Molecular dynamics simulations of the experimentally solved complexes (3 x 500 ns) confirm the differential contact properties with the ECL2. Lisuride shows higher accumulated contact frequencies for C227, L228, and L229 in the ECL2 compared to LSD.

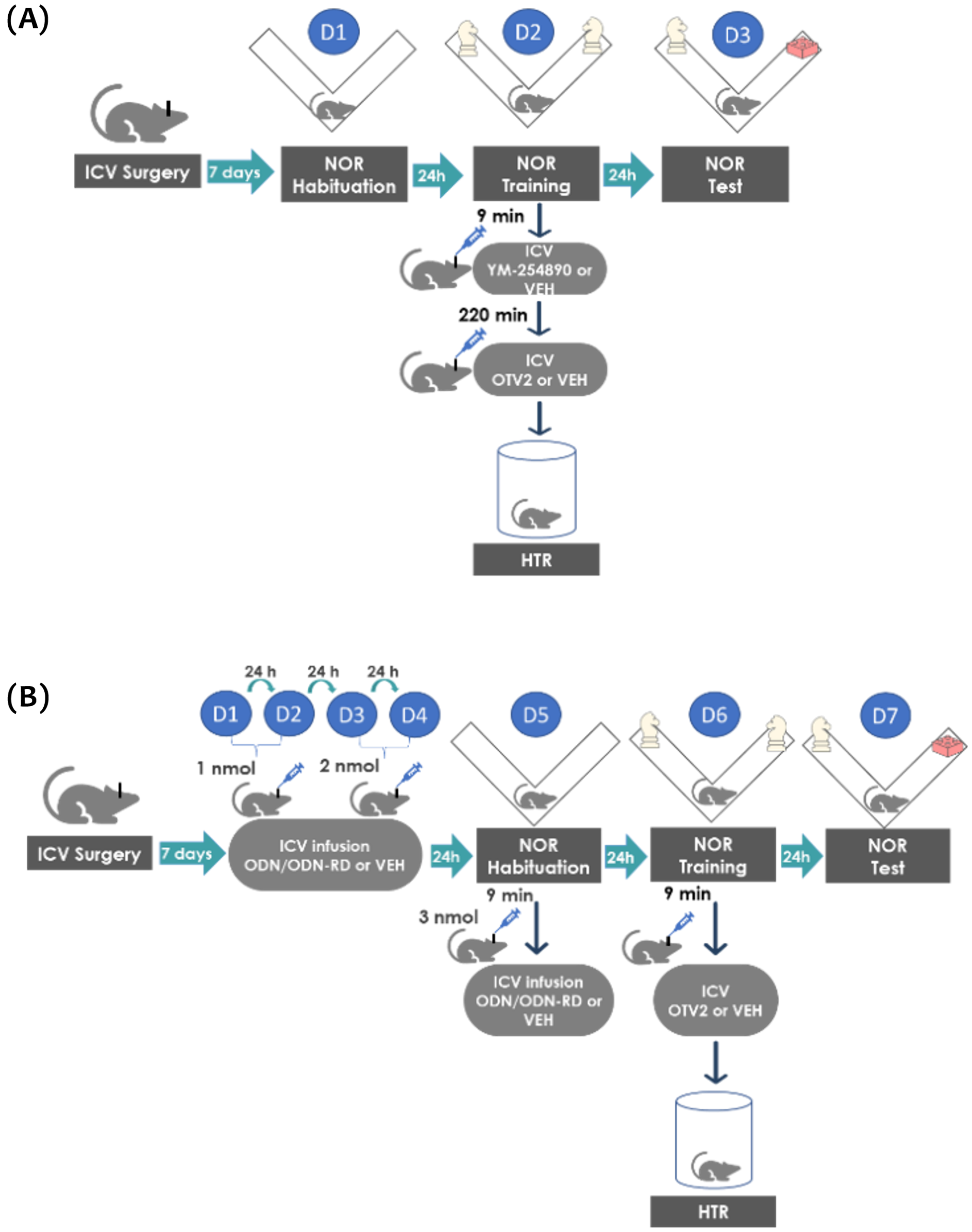

Figure S7. Experimental procedures involved in the central inhibition of specific G proteins and their effects on the HTR, and memory deficits induced by OTV2. (A) To evaluate the role of $G_{\alpha q/11}$ on HTR and long-term memory deficits induced by OTV2, mice were first implanted with ICV cannula. After recovery, they were habituated (day 1) and trained (day 2) to the NOR apparatus. Immediately after training, they received an ICV infusion of the $G_{\alpha q/11}$ inhibitor YM-254890 (16 μM) or vehicle, and 220 min later, ICV infusions of OTV2 or vehicle were performed and HTR was measured for 30 min. The following day, the NOR test was performed. (B) To evaluate the role of $G_{\alpha i1}$ and $G_{\alpha i3}$ on the effects of OTV2, mice received ICV infusions of specific $G_{\alpha i1}$ and $G_{\alpha i3}$ ODNs, ODN-RD or vehicle using the following schedule: on days 1 and 2 mice received 1 nmol, on days 3 and 4 they received 2 nmols, and on day 5 they received 3 nmols. On day 5 (before the ICV infusions), mice were habituated in the NOR. On

day 6, mice were first trained in the NOR and then received ICV infusions of OTV2 or vehicle, and the HTR was measured for 30 min. On day 7, the NOR test was performed.

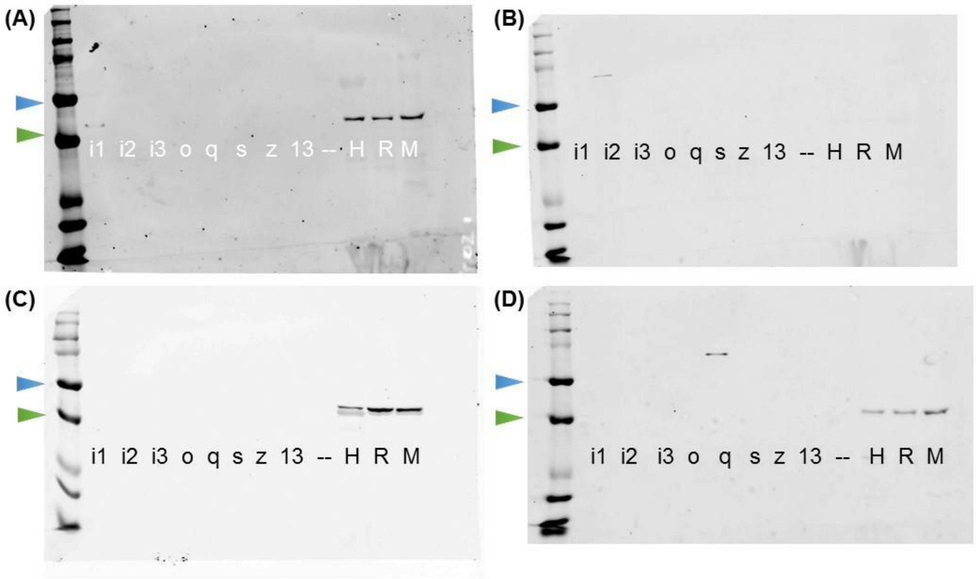

Figure S8. Representative Western-blot images of the selective immunoreactive signal of anti-Gα protein subunit subtypes against recombinant Gα proteins, and against human, rat and mouse brain membrane homogenates samples. Representative Western-blot images of the selective immunoreactive signal of **(A)** anti-$G_{\alpha i1}$ (Santa Cruz Biotechnology Cat# sc-56536), **(B)** anti-$G_{\alpha i2}$ (Santa Cruz Biotechnology Cat# sc-13534), **(C)** anti-$G_{\alpha i3}$ (Antibodies on-line Cat#ABIN6258933), and **(D)** anti-$G_{\alpha q/11}$ (Santa Cruz Biotechnology Cat# sc-515689, RRID:AB_2940775) mouse monoclonal antibodies against different recombinant Gα proteins. Immunodetection of GNAI1 (His-tagged) (Abeomics Cat# 32-3896; lane 2), GNAI2 (GST-tagged) (Antibodies on-line Cat# ABIN1355337; lane 3), GNAI3 (His-tagged) (Abeomics Cat# 32-3898; lane 4), GNAO (His-tagged) (Antibodies on-line Cat# ABIN5709596; lane 5), GNAQ (His-tagged) (Antibodies on-line Cat# ABIN1355345; lane 6), GNAS (GST-tagged) (Antibodies on-line Cat# ABIN1355349; lane 7), GNAZ (His-tagged) (Cusabio Cat# CSB-EP009601HU; lane 8), and GNA13 (His-tagged) (Cusabio Cat# CSB-EP618885HU; lane 9) recombinant proteins, and human (H), rat (R) and mouse (M) brain cortex membranes is shown. Recombinant proteins were purchased from Abeomics (USA), Cusabio (USA) and Antibodies on-line (Germany). MW: Precision Plus Protein Dual Color Standards molecular weight marker (BioRad). Blue arrow head: 50 kDa. Green arrow head: 37 kDa.

(A)

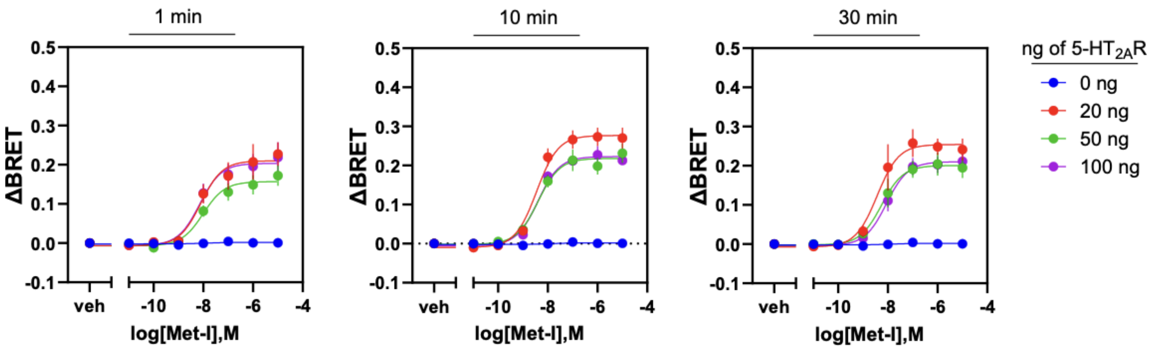

(B)

	1 min			10 min			30 min		
	**pEC50**	**EC50**	**Emax**	**pEC50**	**EC50**	**Emax**	**pEC50**	**EC50**	**Emax**
**0 ng**	7,90	1,26E-08	0,00	7,90	1,26E-08	0,00	7,90	1,26E-08	0,00
**20 ng**	8,10	7,86E-09	0,22	8,45	3,52E-09	0,29	8,44	3,67E-09	0,26
**50 ng**	8,01	9,78E-09	0,16	8,39	4,05E-09	0,22	8,26	5,49E-09	0,20
**100 ng**	8,15	7,01E-09	0,21	8,40	3,98E-09	0,23	8,06	8,68E-09	0,21

Figure S9. The potency of Met-I is not affected by the quantity of receptor expressed. (A) Cells were transfected with an increasing amount of plasmid encoding for the 5-HT$_{2A}$R. Dose-response curves were obtained after stimulation with Met-I as indicated time, to monitor G$_{\alpha q}$ activation using the p63RhoGEF-RlucII sensor and the rGFP-CAAX plasma membrane marker. (B) Table of potency (pEC50) and efficacy (Emax) obtained by BRET assays. Data are represented as ligand-promoted BRET (ΔBRET) as mean ± SEM (n=3).

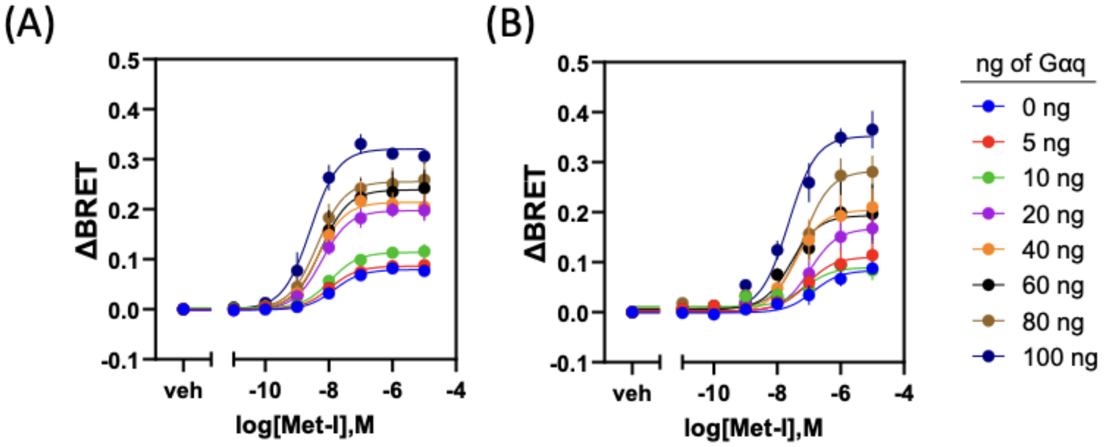

(C)

	HEK SL		CHO	
	**pEC50**	**EC50**	**pEC50**	**EC50**
**0 ng**	7,84	1,44E-08	6,87	1,34E-07
**5 ng**	7,96	1,09E-08	7,06	8,63E-08
**10 ng**	7,95	1,12E-08	7,11	7,71E-08
**20 ng**	8,22	6,03E-09	6,97	1,06E-07
**40 ng**	8,34	4,57E-09	7,44	3,65E-08
**60 ng**	8,25	5,62E-09	7,56	2,71E-08
**80 ng**	8,34	4,58E-09	7,19	6,43E-08
**100 ng**	8,58	2,65E-09	7,64	2,26E-08

Figure S10. Impact of $G_{\alpha q}$ level expression on $5HT_{2A}$R-mediated activation of $G_{\alpha q}$ protein in response to Met-I. HEK293 SL (A) or CHO (B) cells transfected with no (endogenous $G_{\alpha q/11}$) or increasing quantity of $G_{\alpha q}$ protein along with the Gαq activation BRET-based sensor (p63RhoGEF-RlucII) with the plasma membrane marker (rGFP-CAAX) were stimulated with increasing concentrations of Met-I for 10 minutes. (C) Table of pEC50 and EC50 of $G_{\alpha q}$ activation obtained by BRET in HEK or CHO cells. Data are represented as ligand-promoted BRET (ΔBRET) as mean ± SEM (n=3).

6. Supplemental Tables

Table S1. Normalized [^{35}S]GTPγS binding values for $G_{\alpha i1}$, $G_{\alpha i2}$, $G_{\alpha i3}$, and $G_{\alpha q/11}$ in brain cortex membrane homogenates from wild type (WT) and 5-HT_{2A}R KO animals (KO).

	Nitro-I WT					Nitro-I + KO					
	Mean	±	SEM	n	p value	Mean	±	SEM	n	p value	*p value*
Gαi1	113.3	±	3.6	5	**0.022**	102.2	±	2.0	5	ns	***0.037***
Gαi2	102.0	±	2.4	4	ns	104.2	±	1.9	4	ns	*ns*
Gαi3	133.5	±	3.0	3	**0.008**	126.0	±	4.7	3	**0.031**	*ns*
Gαq/11	115.9	±	3.2	5	**0.008**	91.7	±	3.2	5	ns	***0.001***

	Met-I WT					Met-I + KO					
	Mean	±	SEM	n	p value	Mean	±	SEM	n	p value	*p value*
Gαi1	85.6	±	2.7	3	**0.034**	100.9	±	3.2	3	ns	**0.024**
Gαi2	95.3	±	1.3	3	ns	95.3	±	1.3	3	ns	ns
Gαi3	123.6	±	2.3	3	**0.009**	100.1	±	2.5	3	ns	**0.002**
Gαq/11	122.1	±	4.0	3	**0.032**	100.3	±	2.3	3	ns	**0.016**

	Otava 3575001 WT					Otava 3575001 + KO					
	Mean	±	SEM	n	p value	Mean	±	SEM	n	p value	*p value*
Gαi1	88.1	±	3.5	5	**0.026**	100.0	±	1.7	5	ns	***0.022***
Gαi2	124.6	±	4.0	4	**0.009**	116.2	±	2.6	4	**0.009**	*ns*
Gαi3	120.0	±	1.0	3	**0.003**	103.3	±	4.0	3	ns	***0.046***
Gαq/11	121.1	±	2.4	5	**0.001**	101.1	±	2.7	5	ns	***<0.001***

	Otava 3736689 WT					Otava 3736689 + KO					
	Mean	±	SEM	n	p value	Mean	±	SEM	n	p value	*p value*
Gαi1	111.7	±	3.3	5	**0.025**	101.4	±	2.6	5	ns	***0.043***
Gαi2	126.2	±	3.7	4	**0.006**	121.4	±	0.9	4	**<0.001**	*ns*
Gαi3	130.2	±	6.5	3	**0.044**	101.8	±	3.6	3	ns	***0.030***
Gαq/11	134.5	±	4.0	5	**0.001**	95.6	±	2.0	5	ns	***<0.001***

[^{35}S]GTPγS binding values obtained from SPA assays for the different subtypes of G proteins (Gαi1, Gαi2, Gαi3 and Gαq/11), in the presence of a single submaximal concentration of Nitro-I, Met-I, OTV1 (3575001) or OTV2 (3736689) in brain cortex membrane homogenates from wild type animals (WT) and 5-HT2AR KO animals (KO).Specific binding values were transformed to the percentage of basal [^{35}S]GTPγS binding (binding values in the absence of any exogenous drug) obtained for each Gα-protein and considered as 100%. An agonist behavior would result in a significant increase over basal binding, while an inverse agonist would reduce it and an antagonist would not modify it. Results were analyzed by two-tailed Student's t-test (one-sample) vs basal values (expressed as 100%) or by two-tailed Student's t-test (two-sample) between conditions (presence vs absence of MDL-11,939; italicized p values). p values under 0.05 are highlighted in bold. Data are described as mean ± SEM values. ns: non-significant.

Table S2. Description of antibodies and incubation conditions used in antibody-capture [^{35}S]GTPγS binding scintillation proximity assays for the different G_α subunit subtypes.

Target	Antibody	Catalog Number	Concentration	Distributor	[GDP]	Dilution
$G_{\alpha i1}$	Mouse monoclonal anti-$G_{\alpha i1}$	sc-56536	0.2 mg/ml	Santa Cruz	100 µM	1:20
$G_{\alpha i2}$	Mouse monoclonal anti-$G_{\alpha i2}$	sc-13534	0.2 mg/ml	Santa Cruz	50 µM	1:20
$G_{\alpha i3}$	Rabbit polyclonal anti-$G_{\alpha i3}$	ABIN6258933	1 mg/ml	Antibodies on-line	100 µM	1:60
$G_{\alpha q/11}$	Mouse monoclonal anti-$G_{\alpha q/11}$	sc-515689	0.2 mg/ml	Santa Cruz	50 µM	1:20

Table S3. Statistical values obtained in behavioral studies

Experiment	***Drug***	***Behavioural Test***		***Statistical value***
**Dose-response (Two-Tailed Unpaired t test)**	DOI	HTR		t(22)=8.77, p=0.0000
Experiment	***Drug***	***Behavioural Test***	***Effect***	***Statistical value***
**Dose-response (One-way ANOVA)**	OTV2	HTR	Treatment	F(4,41)=4.23, p=0.0059
	Nitro-I	HTR	Treatment	F(4,48)=26.86, p=0.0000
**5-HT2AR KO and WT (Two-way ANOVA)**	OTV1	NOR	Genotype /Treatment	F(2,26)=3.40, p=0.0486
			Genotype	F(1,26)=24.94, p=0.0000
			Treatment	F(2,26)=5.32, p=0.0116
	Met-I	NOR	Genotype /Treatment	F(2,24)=4.32, p=0.0250
			Genotype	F(1,24)=28.95, p=0.0000
			Treatment	F(2,24)=12.06, p=0.0002
	OTV2	HTR	Genotype /Treatment	F(1,15)=9.10, p=0.0087
			Genotype	F(1,15)=5.46, p=0.0338
			Treatment	F(1,15)=24.70, p=0.0002
		NOR	Genotype /Treatment	F(2,25)=3.52, p=0.0449
			Genotype	F(1,25)=6.17, p=0.0200
			Treatment	F(2,25)=5.64, p=0.0095
	Nitro-I	HTR	Genotype /Treatment	F(1,18)=5.20, p=0.0349
			Treatment	F(1,18)=7.15, p=0.0155
**Gαq/11 (Two-way ANOVA)**	OTV2	HTR	Treatment	F(2,27)=35.20, p=0.0000
		NOR	Inhibitor/Treatment	F(2,27)=5.78, p=0.0081
			Inhibitor	F(1,27)=8.50, p=0.0071
**Gαi1 and Gαi3 (Two-way ANOVA)**	OTV2	HTR	Inhibitor/Treatment	F(6,52)=2.29, p=0.0493
			Inhibitor	F(3,52)=6.28, p=0.0010
			Treatment	F(2,52)=12.62, p=0.0000
		NOR	Treatment	F(2,57)=56.88, p=0.0000

7. References

- Abbas AI, Yadav PN, Yao WD, Arbuckle MI, Grant SG, Caron MG, Roth BL. PSD-95 is essential for hallucinogen and atypical antipsychotic drug actions at serotonin receptors. J Neurosci. 2009 Jun 3;29(22):7124-36. doi: 10.1523/JNEUROSCI.1090-09.2009. PMID: 19494135; PMCID: PMC2836830.
- Catlow BJ, Song S, Paredes DA, Kirstein CL, Sanchez-Ramos J. Effects of psilocybin on hippocampal neurogenesis and extinction of trace fear conditioning. Exp Brain Res. 2013 Aug;228(4):481-91. doi: 10.1007/s00221-013-3579-0.
- Diez-Alarcia R, Ibarra-Lecue I, Lopez-Cardona ÁP, Meana J, Gutierrez-Adán A, Callado LF, Agirregoitia E, Urigüen L. Biased Agonism of Three Different Cannabinoid Receptor Agonists in Mouse Brain Cortex. Front Pharmacol. 2016 Nov 4;7:415. doi: 10.3389/fphar.2016.00415.
- Diez-Alarcia R, Muguruza C, Rivero G et al. Opposite alterations of 5-HT2A receptor brain density in subjects with schizophrenia: relevance of radiotracers pharmacological profile. Transl Psychiatry 11, 302 (2021). doi.org/10.1038/s41398-021-01430-7
- Ibarra-Lecue I, Diez-Alarcia R, Morentin B, Meana JJ, Callado LF, Urigüen L. Ribosomal Protein S6 Hypofunction in Postmortem Human Brain Links mTORC1-Dependent Signaling and Schizophrenia. Front Pharmacol 11, 344 (2020). doi: 10.3389/fphar.2020.00344.
- Kenakin T, Christopoulos A. Signalling bias in new drug discovery: detection, quantification and therapeutic impact. Nat Rev Drug Discov. 2013 Mar;12(3):205-16. doi: 10.1038/nrd3954.
- Meneses, A. Stimulation of 5-HT1A, 5-HT1B, 5-HT2A/2C, 5-HT3 and 5-HT4 receptors or 5-HT uptake inhibition: Short- and long-term memory. *Behav. Brain Res.* **184**, 81–90 (2007).
- Nagi K, Pineyro G. Practical guide for calculating and representing biased signaling by GPCR ligands: A stepwise approach. Methods. 2016 Jan 1;92:78-86. doi: 10.1016/j.ymeth.2015.09.010.
- Rambousek L, Palenicek T, Vales K, Stuchlik A. The effect of psilocin on memory acquisition, retrieval, and consolidation in the rat. Front Behav Neurosci. 2014 May 16;8:180. doi: 10.3389/fnbeh.2014.00180.
- Rodriguiz RM, Nadkarni V, Means CR, Pogorelov VM, Chiu YT, Roth BL, Wetsel WC. LSD-stimulated behaviors in mice require β-arrestin 2 but not β-arrestin 1. Sci Rep. 2021 Sep 3;11(1):17690. doi: 10.1038/s41598-021-96736-3.
- Schmid, C. L., Raehal, K. M. & Bohn, L. M. Agonist-directed signaling of the serotonin 2A receptor depends on beta-arrestin-2 interactions in vivo. Proc. Natl. Acad. Sci. U. S. A. 105, 1079–1084 (2008).
- Schmid CL, Bohn LM. Serotonin, but not N-methyltryptamines, activates the serotonin 2A receptor via a ß-arrestin2/Src/Akt signaling complex in vivo. J Neurosci. 2010 Oct 6;30(40):13513-24. doi: 10.1523/JNEUROSCI.1665-10.2010.
- Smith JS, Pack TF, Inoue A, Lee C, Zheng K, Choi I, Eiger DS, Warman A, Xiong X, Ma Z, Viswanathan G, Levitan IM, Rochelle LK, Staus DP, Snyder JC, Kahsai AW, Caron MG, Rajagopal S. Noncanonical scaffolding of Gαi and β-arrestin by G protein-coupled receptors. Science. 2021 Mar 12;371(6534):eaay1833. doi: 10.1126/science.aay1833.

- Urigüen L, García-Fuster MJ, Callado LF, Morentin B, La Harpe R, Casadó V, Lluis C, Franco R, García-Sevilla JA, Meana JJ. Immunodensity and mRNA expression of A2A adenosine, D2 dopamine, and CB1 cannabinoid receptors in postmortem frontal cortex of subjects with schizophrenia: effect of antipsychotic treatment. Psychopharmacology (Berl). 2009 Oct;206(2):313-24. doi: 10.1007/s00213-009-1608-2.
- Zhang, G. & Stackman, R. W. The role of serotonin 5-HT2A receptors in memory and cognition. Front. Pharmacol. 6, (2015).
- Zhang G, Ásgeirsdóttir HN, Cohen SJ, Munchow AH, Barrera MP, Stackman RW Jr. Stimulation of serotonin 2A receptors facilitates consolidation and extinction of fear memory in C57BL/6J mice. Neuropharmacology. 2013 Jan;64(1):403-13. doi: 10.1016/j.neuropharm.2012.06.007.
