# Supplementary material for: G protein-specific mechanisms in the serotonin 5-HT2A receptor regulate psychosis-related effects and memory deficits
Source: Nat Commun. 2024 May 29;15:4307. doi: 10.1038/s41467-024-48196-2 (PMC11137019; doi:10.1038/s41467-024-48196-2)
Supplement: Supplementary file 3 — Reporting Summary [file 41467_2024_48196_MOESM3_ESM.pdf]

Corresponding author(s): Jana Selent and Patricia Robledo

Last updated by author(s): March 12, 2023

Reporting Summary

Nature Portfolio wishes to improve the reproducibility of the work that we publish. This form provides structure for consistency and transparency in reporting. For further information on Nature Portfolio policies, see our Editorial Policies and the Editorial Policy Checklist.

Statistics

For all statistical analyses, confirm that the following items are present in the figure legend, table legend, main text, or Methods section.

n/a	Confirmed	
☐	☒	The exact sample size (*n*) for each experimental group/condition, given as a discrete number and unit of measurement
☐	☒	A statement on whether measurements were taken from distinct samples or whether the same sample was measured repeatedly
☐	☒	The statistical test(s) used AND whether they are one- or two-sided<br>*Only common tests should be described solely by name; describe more complex techniques in the Methods section.*
☐	☒	A description of all covariates tested
☐	☒	A description of any assumptions or corrections, such as tests of normality and adjustment for multiple comparisons
☐	☒	A full description of the statistical parameters including central tendency (e.g. means) or other basic estimates (e.g. regression coefficient) AND variation (e.g. standard deviation) or associated estimates of uncertainty (e.g. confidence intervals)
☐	☒	For null hypothesis testing, the test statistic (e.g. *F*, *t*, *r*) with confidence intervals, effect sizes, degrees of freedom and *P* value noted<br>*Give P values as exact values whenever suitable.*
☒	☐	For Bayesian analysis, information on the choice of priors and Markov chain Monte Carlo settings
☒	☐	For hierarchical and complex designs, identification of the appropriate level for tests and full reporting of outcomes
☒	☐	Estimates of effect sizes (e.g. Cohen's *d*, Pearson's *r*), indicating how they were calculated

Our web collection on statistics for biologists contains articles on many of the points above.

Software and code

Policy information about availability of computer code

Data collection	1. Structural models were obtained with the MODELLER Version 10.0.<br>2. Molecular dynamics data of the structural models were collected using the ACEMD3 simulation engine.
Data analysis	1. The statistical analyses were performed with the “Statistica” programme, version 6 (StatSoft Inc.) and GraphPad PrismTM.<br>2. Cpptraj has been used for processing coordinate trajectories and data files.

For manuscripts utilizing custom algorithms or software that are central to the research but not yet described in published literature, software must be made available to editors and reviewers. We strongly encourage code deposition in a community repository (e.g. GitHub). See the Nature Portfolio guidelines for submitting code & software for further information.

Data

Policy information about availability of data

All manuscripts must include a data availability statement. This statement should provide the following information, where applicable:

- Accession codes, unique identifiers, or web links for publicly available datasets
- A description of any restrictions on data availability
- For clinical datasets or third party data, please ensure that the statement adheres to our policy

Data supporting the findings of this manuscript are available as a Supplementary Information file. MD simulations are deposited at the GPCRmd database

(www.gpcrmd.org).
Met-I bound to 5-HT2AR: https://submission.gpcrmd.org/view/1105/
Nitro-I bound to 5-HT2AR: https://submission.gpcrmd.org/view/1107/
OTV1 bound to 5-HT2AR: https://submission.gpcrmd.org/view/1128/
OTV2 bound to 5-HT2AR: https://submission.gpcrmd.org/view/1110/
LSD bound to 5-HT2AR (PDB 6WGT): https://submission.gpcrmd.org/view/1175/
Lisuride bound to 5-HT2AR (PDB 7WC7): https://submission.gpcrmd.org/view/1176/
Additional data supporting the findings are available from the corresponding authors upon reasonable request.

Research involving human participants, their data, or biological material

Policy information about studies with human participants or human data. See also policy information about sex, gender (identity/presentation), and sexual orientation and race, ethnicity and racism.

Reporting on sex and gender	Samples from both male and female human post-mortem brains were used.
Reporting on race, ethnicity, or other socially relevant groupings	The age of the participants was between 29–90 years, and the postmortem delay between death and storage of the samples ranged from 4 to 12 h, and the storage time between sampling and experiments ranged from 48 to 10 months.
Population characteristics	All the subjects were determined to be free of neurological and psychiatric disorders based on medical records and postmortem tissue examinations. Positive blood toxicology for drugs or ethanol was considered exclusion criteria. Samples from the dorsolateral prefrontal cortex (PFC) were dissected at autopsy following established protocols55
Recruitment	Human brain samples were obtained at autopsy in the Basque Institute of Legal Medicine, Bilbao, Spain.
Ethics oversight	Human brain samples were manipulated in compliance with policies of research from the ethical board of Basque Institute of Legal Medicine, Bilbao, Spain.

Note that full information on the approval of the study protocol must also be provided in the manuscript.

Field-specific reporting

Please select the one below that is the best fit for your research. If you are not sure, read the appropriate sections before making your selection.

☒ Life sciences ☐ Behavioural & social sciences ☐ Ecological, evolutionary & environmental sciences

For a reference copy of the document with all sections, see nature.com/documents/nr-reporting-summary-flat.pdf

Life sciences study design

All studies must disclose on these points even when the disclosure is negative.

Sample size	Sample sizes were determined according to previous studies from our laboratories and from similar published work in the literature.<br>1. memory testing: Viñals, X. Cognitive Impairment Induced by Delta9-tetrahydrocannabinol Occurs through Heteromers between Cannabinoid CB1 and Serotonin 5-HT2A Receptors.<br>2. HTR testing: González-Maeso, J. Transcriptome fingerprints distinguish hallucinogenic and nonhallucinogenic 5-hydroxytryptamine 2A receptor agonist effects in mouse somatosensory cortex.
Data exclusions	Regarding the postmortem brain samples, subjetcs with positive blood toxicology for drugs or ethanol were excluded from the study.
Replication	In cell based studies, replicates of 3 were performed and they were all successful<br>In ex-vivo studies, n=12 was used. 3 to 6 independent experiments were carried out in triplicate, all of them successful<br>In behavioral experiments, n=5-12 mice were used.
Randomization	Randomization is not relevant in cell based studies<br>In ex-vivo studies, prefrontal cortex samples were randomly allocated into experimental groups.<br>Mice were randomized into different experimental groups/conditions according to weight and age.
Blinding	Researchers were blind to group allocation in the different experimental contitions and to data collection.

Reporting for specific materials, systems and methods

We require information from authors about some types of materials, experimental systems and methods used in many studies. Here, indicate whether each material, system or method listed is relevant to your study. If you are not sure if a list item applies to your research, read the appropriate section before selecting a response.

Materials & experimental systems

n/a	Involved in the study
☐	☒ Antibodies
☐	☒ Eukaryotic cell lines
☒	☐ Palaeontology and archaeology
☐	☒ Animals and other organisms
☒	☐ Clinical data
☒	☐ Dual use research of concern
☒	☐ Plants

Methods

n/a	Involved in the study
☒	☐ ChIP-seq
☒	☐ Flow cytometry
☒	☐ MRI-based neuroimaging

Antibodies

Antibodies used

Mouse monoclonal antibodies against Gai1 (sc-56536), Gai2 (sc-13534), and Gaq/11 (sc-515689) proteins were obtained from Santa Cruz Biotechnologies (USA). Rabbit polyclonal anti-Gai3-protein (ABIN6258933) was obtained from Antibodies online.

Validation

The specificity of the antibodies vs the different recombinant G proteins and brain tissue has been previously demonstrated (Diez-Alarcia et al., 2021).

Eukaryotic cell lines

Policy information about cell lines and Sex and Gender in Research

Cell line source(s)

HEK-293 clonal cell lines were a gift from S. Laporte (McGill University, Montreal, Quebec, Canada).
Plasmids encoding β-arrestin1-RlucII36, human GRK237, rGFP-CAAX17,37, and wild-type Gαi2 protein and an effector protein fused with RlucII (Rap1GAP-RlucII)20 were previously described.

Authentication

None of the cell lines used were authenticated.

Mycoplasma contamination

Cells were negative for mycoplasma contamination.

Commonly misidentified lines
(See ICLAC register)

No commonly misidentified cell lines were used.

Animals and other research organisms

Policy information about studies involving animals; ARRIVE guidelines recommended for reporting animal research, and Sex and Gender in Research

Laboratory animals

Male homozygous 5-HT2AR KO mice and WT littermates on a C57BL/6J background at 8 weeks of age (25-30 g) were used.

Wild animals

The study did not involve wild animals.

Reporting on sex

mice experiments were performed in males only.

Field-collected samples

This study did not involve samples collected from the field.

Ethics oversight

Animal procedures were carried out following the standard ethical guidelines (European Communities Directive 86/609 EEC) and approved by the local Parc de Recerca Biomédica de Barcelona (PRBB) ethical committee.

Note that full information on the approval of the study protocol must also be provided in the manuscript.

Plants

Seed stocks

n/a

Novel plant genotypes

n/a

Authentication

n/a
