# Supplementary material for: G protein-specific mechanisms in the serotonin 5-HT2A receptor regulate psychosis-related effects and memory deficits
Source: Nat Commun. 2024 May 29;15:4307. doi: 10.1038/s41467-024-48196-2 (PMC11137019; doi:10.1038/s41467-024-48196-2)
Supplement: Supplementary file 4 — Source Data [file 41467_2024_48196_MOESM4_ESM.zip › Uncropped_Gels.pdf]

Cortex / Cerebelum / “Midbrain”

(pooling of samples)

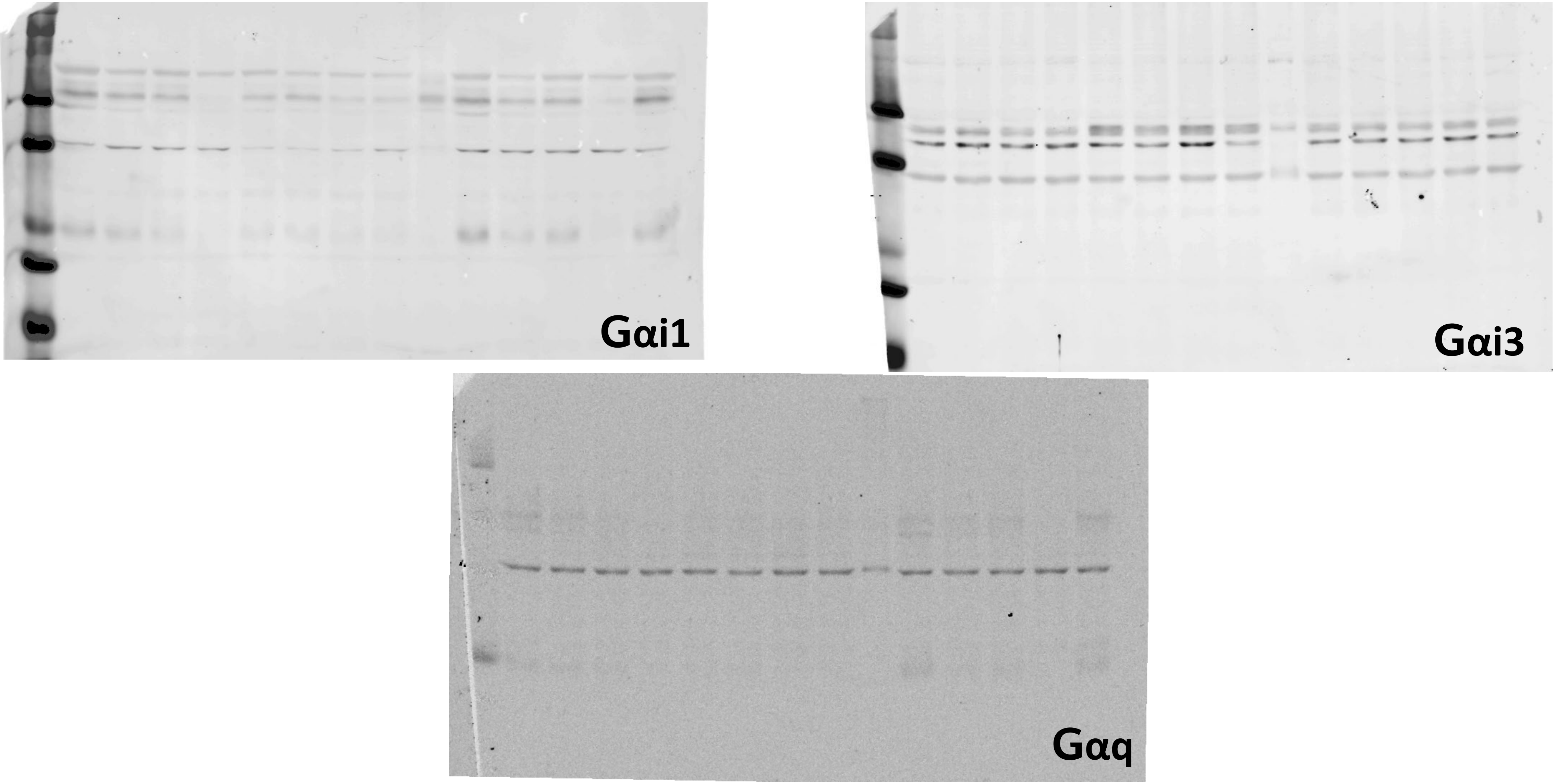

Cortex / Cerebelum / "Midbrain"

(pooling of samples)

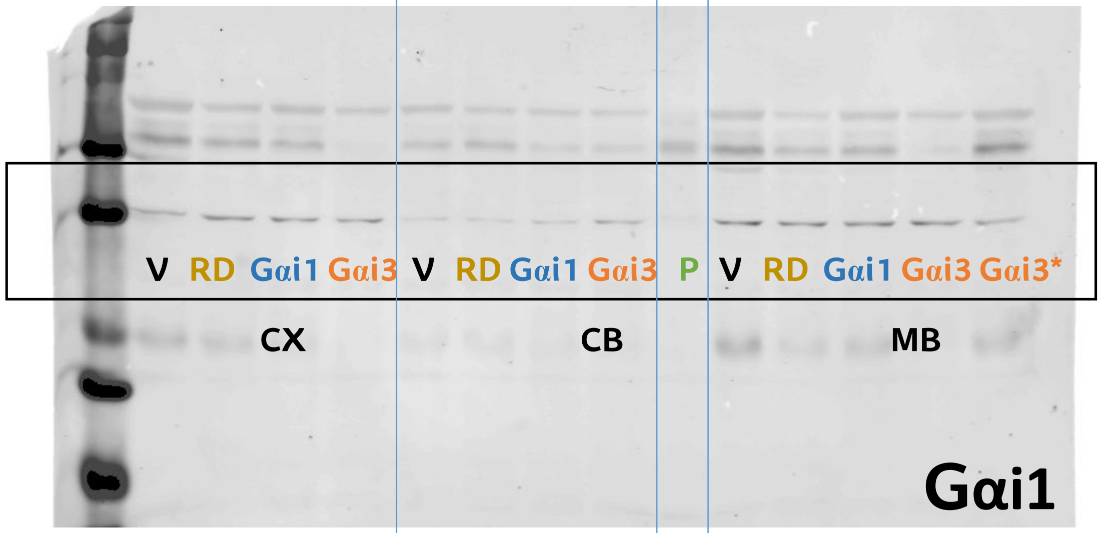

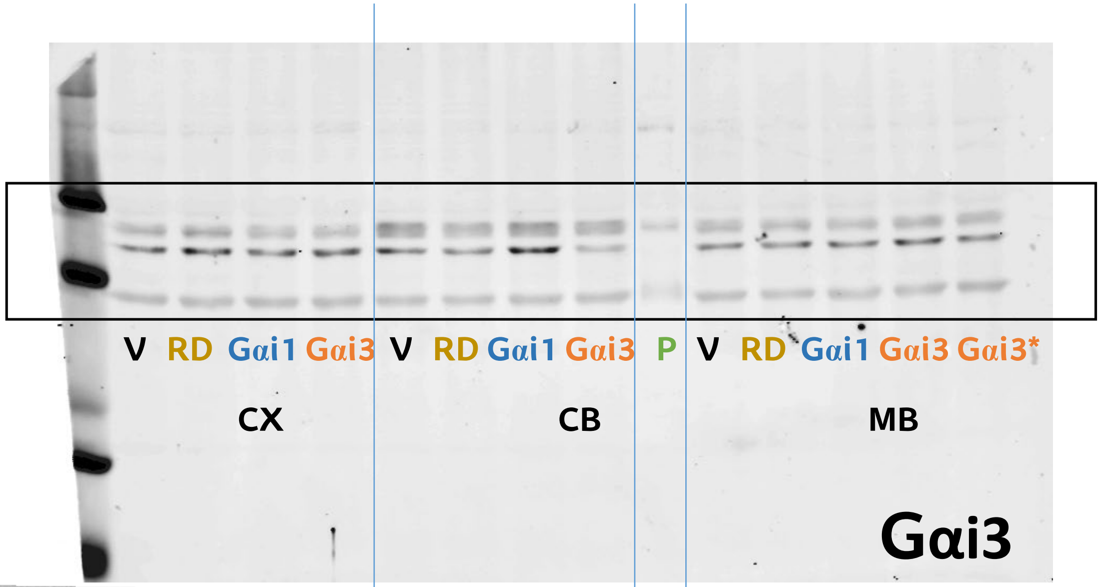

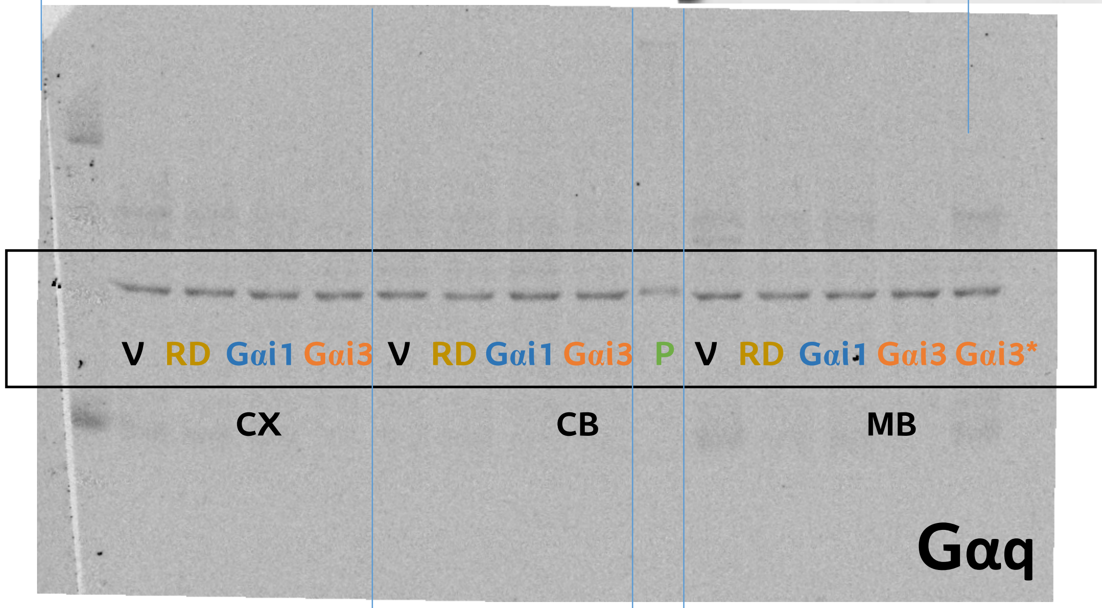

Cortex / Cerebelum / "Midbrain"

(pooling of samples)

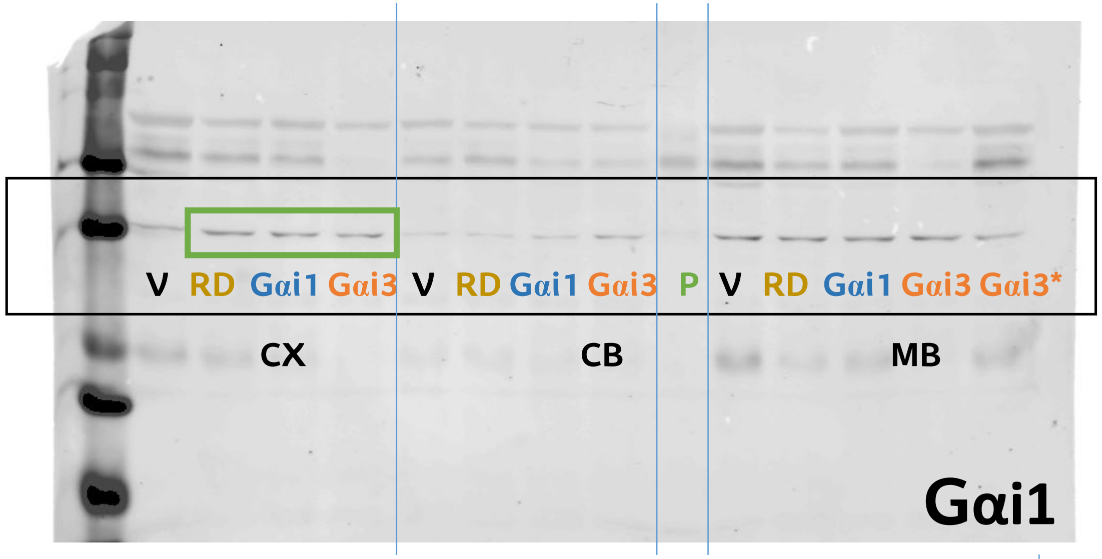

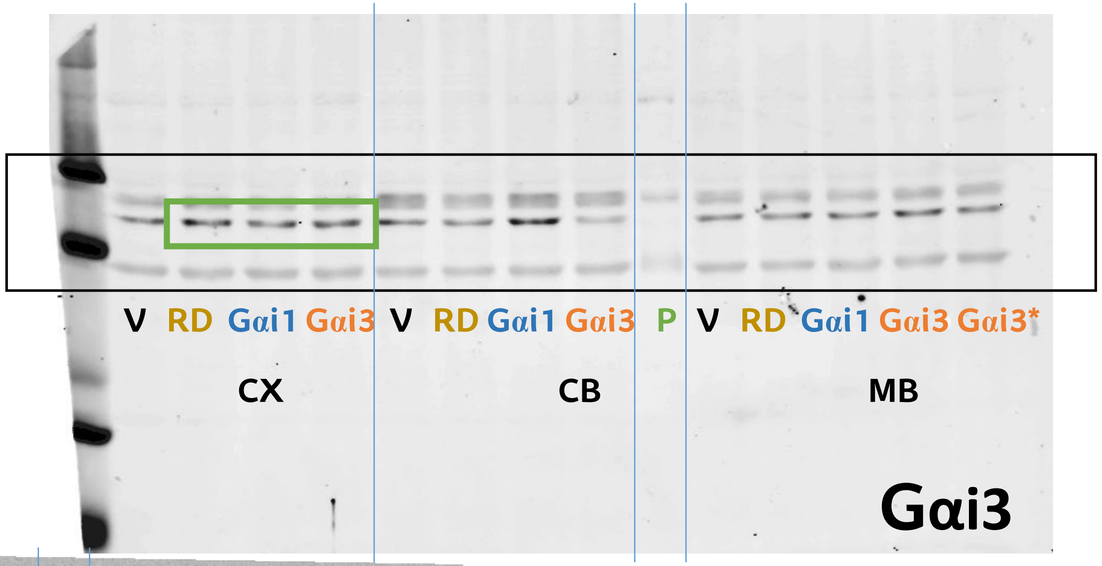

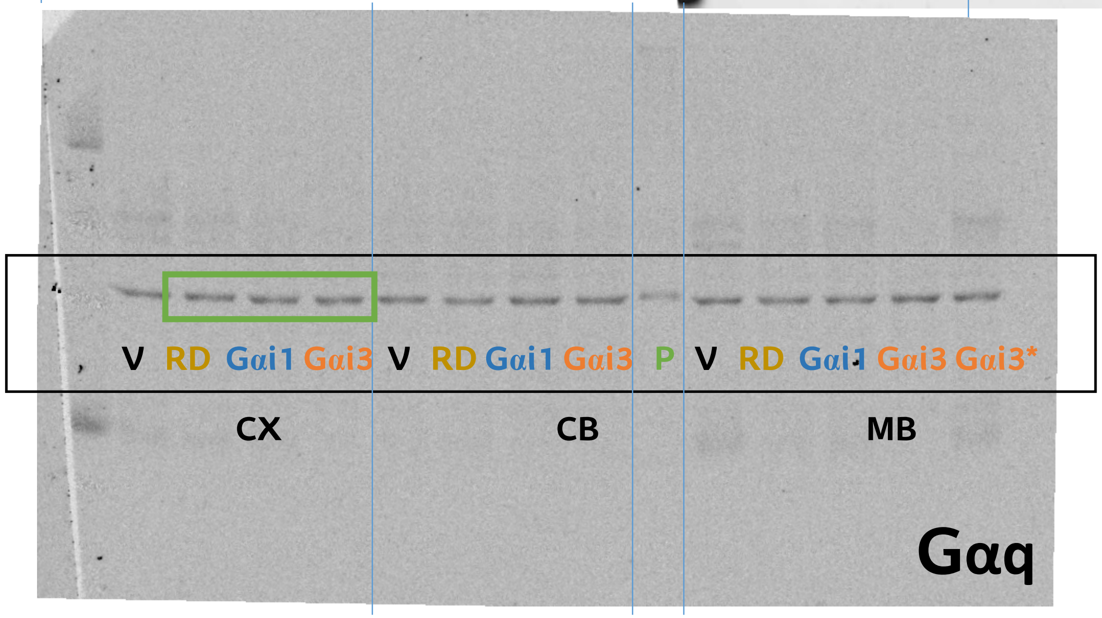

Cortex / Cerebelum / “Midbrain”

(pooling of samples)

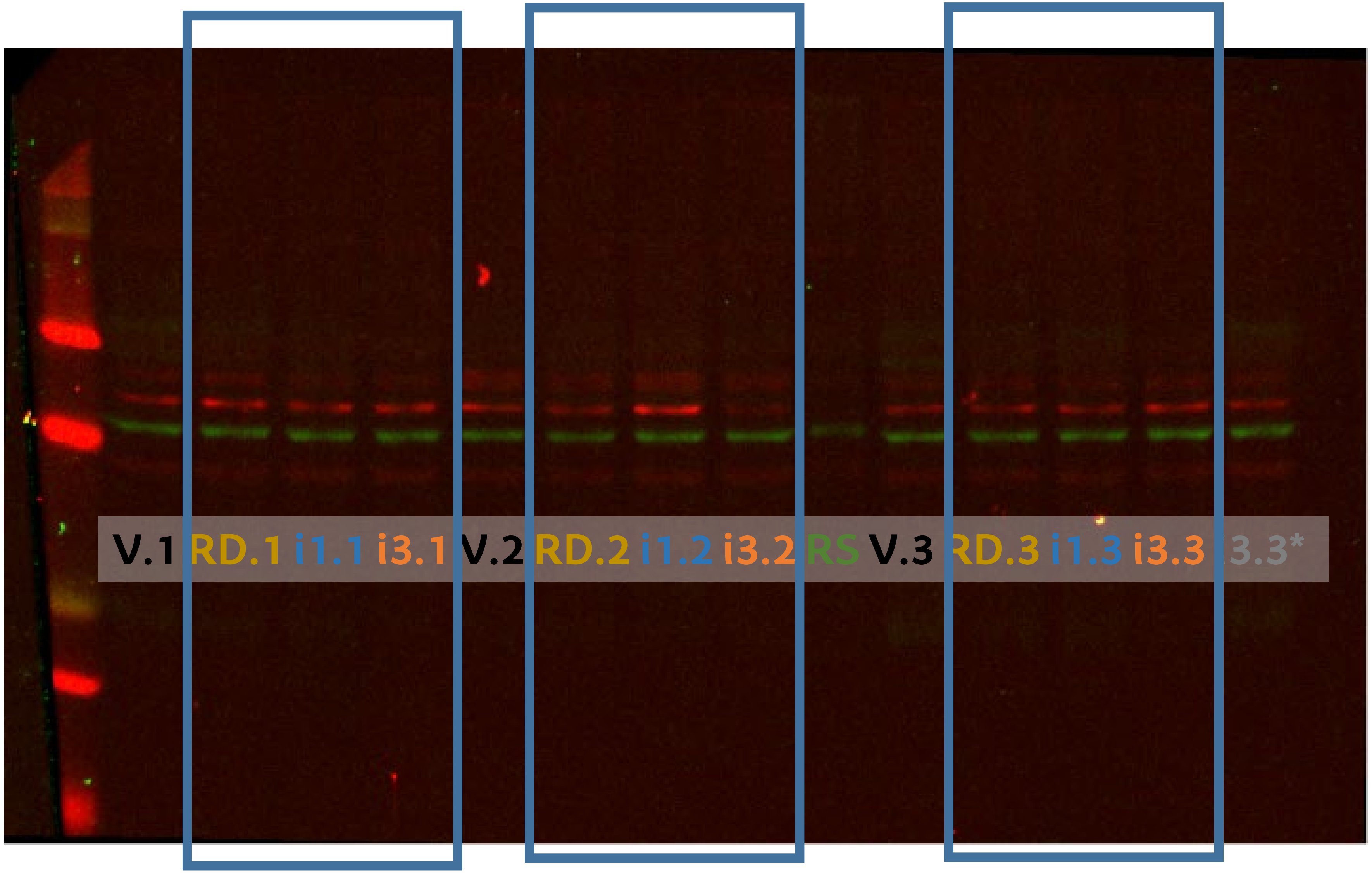

GNAI3

GNAQ
